# A Veterinary DICOM‐Based Deep Learning Denoising Algorithm Can Improve Subjective and Objective Brain MRI Image Quality

**DOI:** 10.1111/vru.70015

**Published:** 2025-02-13

**Authors:** Wilfried Mai, Silke Hecht, Matthew Paek, Shannon P. Holmes, Hugo Dorez, Martin Blanchard, Jamil Nour Eddin

**Affiliations:** ^1^ Department of Clinical Sciences and Advanced Medicine School of Veterinary Medicine University of Pennsylvania Philadelphia Pennsylvania USA; ^2^ Department of Small Animal Clinical Sciences, College of Veterinary Medicine University of Tennessee Knoxville Tennessee USA; ^3^ Synergy Veterinary Imaging Partners MD/VA Frederick Maryland USA; ^4^ AXIS – Animal Cross‐Sectional Imaging Specialists Sarasota Florida USA; ^5^ Hawkcell Lyon France

**Keywords:** artificial intelligence, noise reduction, postprocessing

## Abstract

In this analytical cross‐sectional method comparison study, we evaluated brain MR images in 30 dogs and cats with and without using a DICOM‐based deep‐learning (DL) denoising algorithm developed specifically for veterinary patients. Quantitative comparison was performed by measuring signal‐to‐noise (SNR) and contrast‐to‐noise ratios (CNR) on the same T2‐weighted (T2W), T2‐FLAIR, and Gradient Echo (GRE) MR brain images in each patient (native images and after denoising) in identical regions of interest. Qualitative comparisons were then conducted: three experienced veterinary radiologists independently evaluated each patient's T2W, T2‐FLAIR, and GRE image series. Native and denoised images were evaluated separately, with observers blinded to the type of images they were assessing. For each image type (native and denoised) and pulse sequence type image, they assigned a subjective grade of coarseness, contrast, and overall quality. For all image series tested (T2W, T2‐FLAIR, and GRE), the SNRs of cortical gray matter, subcortical white matter, deep gray matter, and internal capsule were statistically significantly higher on images treated with DL denoising algorithm than native images. Similarly, for all image series types tested, the CNRs between cortical gray and white matter and between deep gray matter and internal capsule were significantly higher on DL algorithm‐treated images than native images. The qualitative analysis confirmed these results, with generally better coarseness, contrast, and overall quality scores for the images treated with the DL denoising algorithm. In this study, this DICOM‐based DL denoising algorithm reduced noise in 1.5T MRI canine and feline brain images, and radiologists’ perceived image quality improved.

## 1 Introduction

MRI scans are time‐consuming due to the multiplicity of pulse sequences obtained in each patient, leading to prolonged anesthesia times. Operators can reduce acquisition times using various strategies, such as decreasing the number of acquisitions and the matrix size, increasing the bandwidth or slice thickness, or acquiring fewer slices with a wider inter‐slice gap. However, these strategies have adverse effects, including reduced signal‐to‐noise ratio (SNR), partial volume averaging, or decreased spatial resolution. Scanners with higher field strength (e.g., 3T) can produce images of similar or higher SNR and resolution in shorter times, but these are costly solutions. For these reasons, many strategies have been developed to try and improve SNR on MRI images, such as postprocessing with filtering or transformation methods. In recent years, deep learning approaches (a subfield of machine learning) for noise removal have been developed in human medical imaging, with better performance than the conventional denoising methods available [1].

Many MRI manufacturers have recently marketed Deep Learning‐based solutions to improve SNR on MR images (e.g., Air Recon DL for GE Healthcare, Deep Resolve for Siemens, AiCE for Canon). However, they are costly solutions based on data processing in k‐space and/or are scanner‐specific. In addition, these algorithms were trained on human patient data. Validated, effective, veterinary‐specific, platform‐agnostic Deep Learning algorithms are currently unavailable to improve MR image quality. Although still in its infancy, there is an accelerating interest in using and developing artificial intelligence (AI) solutions in veterinary diagnostic imaging [2, 3, 4, 5, 6, 7, 8, 9, 10, 11, 12, 13, 14, 15, 16, 17, 18, 19, 20].

This study aimed to evaluate a commercially available denoising deep learning‐based algorithm (HawkAI) explicitly developed by one author (JNE, MSc in image and data processing) for veterinary MRI that functions across all imaging platforms. This algorithm works on DICOM datasets (i.e., image space) instead of the raw data and hence can be applied to many scanners and protocols (i.e., contrasts). We specifically aimed to evaluate 1.5T canine and feline brain images subjected to the HawkAI algorithm both qualitatively and quantitatively and compare them with the original 1.5T images. We hypothesized that conventional 1.5T DICOM MRI images treated with an AI‐based denoising algorithm would provide higher SNR and contrast images than the native images. We also hypothesized that experienced veterinary radiologists’ subjective perception of image quality (coarseness, contrast, and overall image quality) would be higher for AI‐treated images than native images.

## 2 Methods

### 2.1 Study Population

This was an analytical cross‐sectional method comparison study. A convenience sample of 30 consecutive brain MRI examinations of dogs and cats was extracted from the PACS database of the University of Pennsylvania School of Veterinary Medicine. All owners had signed consent forms to use patient data at admission to the hospital. All animals were imaged for clinical reasons using the same clinical MRI scanner (GE Healthcare 1.5T Signa Explorer). The same standardized protocols were used in all animals. They included T2W Sagittal Fast Spin Echo (FSE), Transverse T2W FSE, Transverse T2‐FLAIR, Transverse T2*W Gradient Echo (GRE), DWI series with ADC maps in the transverse plane, T1W Spin Echo in the transverse and dorsal plane pre‐ and post‐Gadolinium administration. Patients were imaged under general anesthesia and in sternal recumbency using a 16‐channel Flexible Coil placed around the head for brain imaging. Before imaging, a brief surface coil sensitivity calibration scan was obtained to correct the nonuniform receiver coil profile (phased‐array uniformity enhancement [PURE]). Exact imaging parameters such as field of view, repetition time (TR), and echo time (TE) varied across examinations depending on the patient's size.

### 2.2 Deep Learning Denoising Algorithm Principles

#### 2.2.1 Training Database

The algorithm training database consisted of clinical MRI scans of normal and diseased cats and dogs acquired from five different veterinary hospitals (three equipped with a GE Healthcare Signa 1.5T scanner, one with a Siemens MAGNETOM Sempra 1.5T, and one with an ESAOTE Vet‐MR Grande 0.25T). During each exam, an additional pulse sequence was acquired with a reduced number of averages (also called Number of Excitations, NEX), creating a pair of high‐quality/noisy images that were then fed to the neural network for training.

A total of 34,841 pairs of high‐quality/noisy images were gathered at the time of the study, covering T1 and T2 contrast (both 2D and 3D sequences) in three imaging planes (transverse, sagittal, and dorsal). The image training database was separated into subsets. Ninety percent was allocated for training and 10% for the test set. The training set was partitioned into 90% for training and 10% for validation. The 30 patients included in this study were not part of the training database used for developing the denoising algorithm.

#### 2.2.2 Model Architecture and Training

HawkAI is a generative adversarial net‐type model [21] with paired two‐dimensional brain and spine MRI images of dogs and cats. A U‐net with a hyperbolic tangent activation layer was used for training, with a hybrid loss function (a combination of perceptual loss [22], pixel‐wise loss, and adversarial loss). Noisy images were used as the input of the network, and high‐quality images were used as the reference images for the loss calculation. Then, the weights of the model were updated using ADAM [23] optimizer with a learning rate set to 10–4. Training images were normalized between 0 and 1, and reference images were normalized between −1 and 1. All images were resized to 512 × 512 if they were not already this size.

The training was done using the TensorFlow/Keras library (version 2.15) in a Python 3.11.7 environment on an NVIDIA Quadro GV100 workstation with 32GB memory. Postprocessing was applied to the denoised image with a technique based on high boost filtering [24] and histogram matching [25] to preserve the original contrast as well as possible while still sharpening the image adequately to counteract the blurring effects of a denoising process.

### 2.3 Quantitative Image Evaluation

**FIGURE 1:**
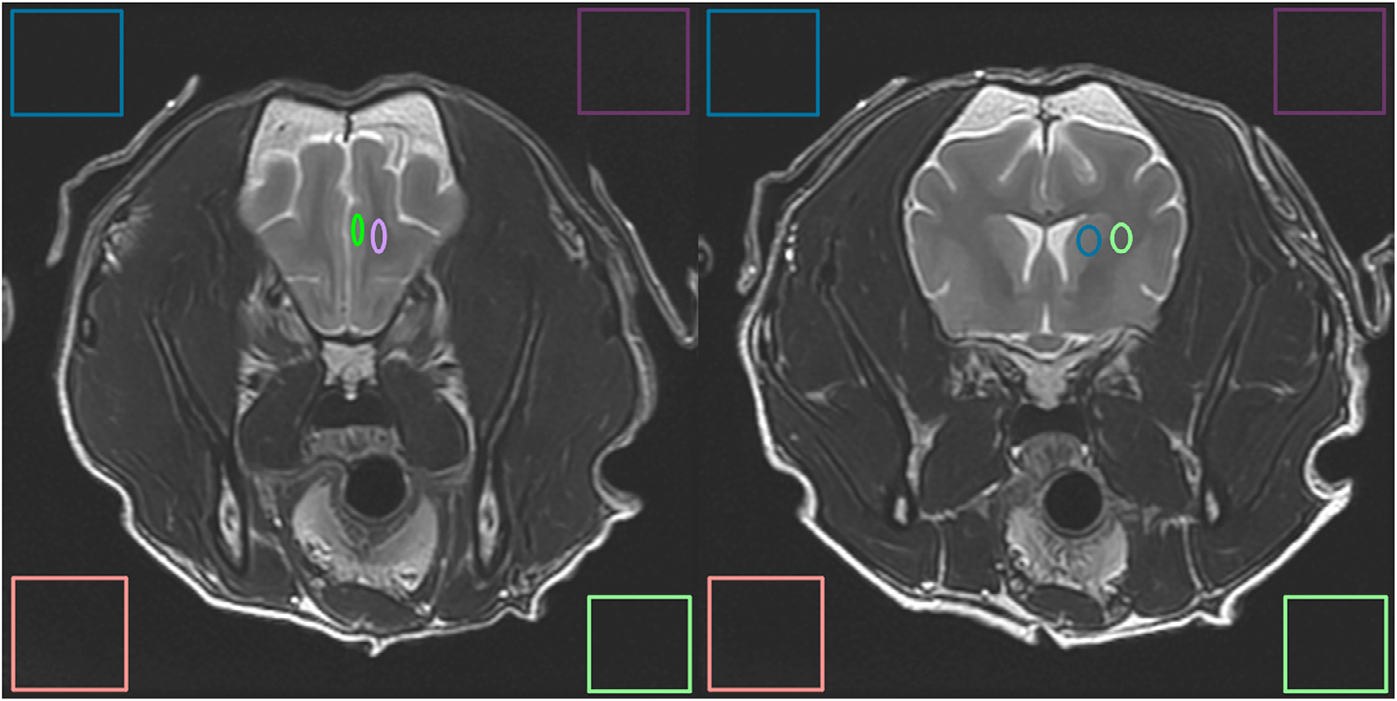
ROI placement for measurement of SNR and CNR (T2W FSE images at the level of the frontal lobes (left) and caudate nuclei (right), as well as ROIs outside of the patient to measure the SD of the background noise signal.

The first author (W. M.), an ACVR and ECVDI board‐certified radiologist, performed measurements for the quantitative analysis by measuring the SNR and contrast‐to‐noise ratio (CNR) on native and AI‐treated images. These measurements were only performed on images deemed free of significant pathology causing mass effects or alterations of signal intensity. Patients were excluded from the quantitative analysis if significant pathology or artifacts were present. Regions of interest (4–8 mm^2^) were first placed on transverse T2W images using the circle/ellipse ROI tool of a standard DICOM viewer (Horos) as follows (Figure 1):

‐ Cortical gray matter (GM) and adjacent white matter (WM) ROIs were placed in the medial aspect of the left or right frontal lobe.
‐ Deep GM ROI was placed over the caudate nucleus, and WM ROI on the same image was placed in the adjacent internal capsule.

Four squared ROIs were placed in the air regions of the field of view to measure the background noise's standard deviation (SD) in the four corners of the image, avoiding regions of artifacts such as phase ghosting (Figure 1).

These ROIs were then placed on T2‐FLAIR and GRE images of the same patient (native and AI‐treated) in the same transverse plane using the propagation tool of the DICOM viewer so that all measurements were obtained at precisely the same level.

The mean signal intensity (SI) of the brain regions and the SD of the background noise were measured; the SD of the background noise in the four ROIs outside of the patient was averaged to obtain the overall SD_background noise_. SNRs and CNRs were then calculated using the following formulas [26, 27]:

$$\mathrm{SN}R_{\mathrm{tissue}}=SI_{\mathrm{tissue}}/SD_{\mathrm{background}\mathrm{noise}}$$

$$\mathrm{CN}{R_{\mathrm{tissue}1}}_{-\mathrm{tissue}2}=\left(SI_{\mathrm{tissue}1}-SI_{\mathrm{tissue}2}\right)/SD_{\mathrm{background}\mathrm{noise}}$$

### 2.4 Subjective Image Evaluation

All DICOM images (native and postprocessed by the denoising algorithm) were assessed by three ACVR board‐certified veterinary radiologists (S. H., M. P., and S. P. H.) with more than ten years of experience interpreting canine and feline MRI studies. For subjective image quality analysis, readers were asked to focus on the transverse T2W FSE, T2‐FLAIR, and GRE image series. These sequences were selected because they typically have the lowest SNR and provide inherent good contrast between the gray and white matter. The 60 sets of DICOM images (30 native and 30 postprocessed) were anonymized, organized in random order, and made available to the observers in a cloud‐based folder. Observers used the DICOM viewer of their choice and were blinded to the type of images (native or AI postprocessed) they were grading. The readers independently evaluated the perceived coarseness of the images (defined as image graininess as a surrogate marker of noise), perceptive contrast between the deep GM structures (e.g. basal nuclei, thalamus), and WM tracks (e.g., corona radiata, internal capsule), perceptive contrast between the cortical gray matter and adjacent white matter, and overall image quality of the images using a four‐point scoring system (Table 1).

**TABLE 1:** Score categories for the radiologists' subjective evaluation of MRI image quality.

Score	Coarseness	Contrast	Overall image quality
0	Severe coarseness is obscuring anatomy	Structures are not separated	Poor
1	Significant coarseness is present but anatomical structures can be distinguished	Minimal contrast	Acceptable
2	Moderate to mild coarseness	Structures are separated with moderate contrast	Good
3	No coarseness	Clear separation between structures with excellent contrast	Excellent

### 2.5 Statistical Analysis

Statistical analyses were performed by the first author (WM), who holds a Clinical Research and Biostatistics certificate. All statistical tests were performed using commercially available software (Stata v.18.0, College Station, TX, USA). The normality of the data distribution was assessed using the Shapiro–Wilk test, where *p*‐values > .05 indicated normal distribution.

The average and 95% confidence intervals of SNRs and CNRs for each measurement and pulse sequence were computed. Qualitative image quality scores (coarseness, contrast, and overall quality) were expressed as median and range.

The SNRs and CNRs were compared between native and AI‐treated images using the one‐sided paired Wilcoxon sign‐ranked test. For each reviewer, the perceived image quality scores assigned to the T2W, T2‐FLAIR, and GRE AI‐treated images were compared with those assigned to the native images using the paired Wilcoxon sign‐ranked test. Differences were considered statistically significant if *p*‐values were < .05.

## 3 Results

### 3.1 Study Group

30 canine and feline brain MRI scans obtained between December 2023 and March 2024 were included.

There were six cats (three male castrated and three female spayed) with a median age of 4 years (range 1.2–10.2). There were four domestic shorthairs, one Norwegian forest cat, and one Russian blue cat.

There were 24 dogs (10 female spayed, one female intact, nine male castrated, and four male intact) with a median age of 7.6 years (range 0.3–14.7). There were nine mixed‐breed dogs, three Labrador retrievers, two Shih Tzus, and one each American Pit Bull terrier, Staffordshire Bull terrier, Maltese, German shepherd, Australian shepherd, French bulldog, standard poodle, Dachshund, Pug, and Jack Russel terrier.

### 3.2 Quantitative Analysis

Three brain MRIs were excluded from quantitative analysis due to artifacts or significant pathology diffusely altering the signal intensity of gray and white matter or distorting anatomy to a degree preventing adequate placement of ROIs. Therefore, 27 brain MRIs were included in this part of the study.

**FIGURE 2:**
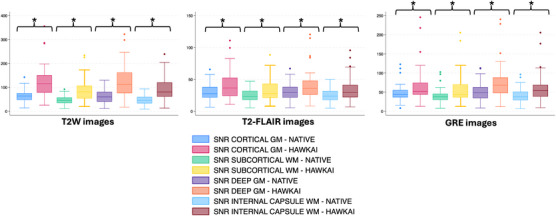
Box and whiskers plots showing the quantitative measurements of SNR on T2W, T2‐FLAIR, and GRE images of 27 brain examinations. GM, Gray matter; WM, White matter. *Statistically significant differences between paired datasets (*p* < .0001 for all tests).

**FIGURE 3:**
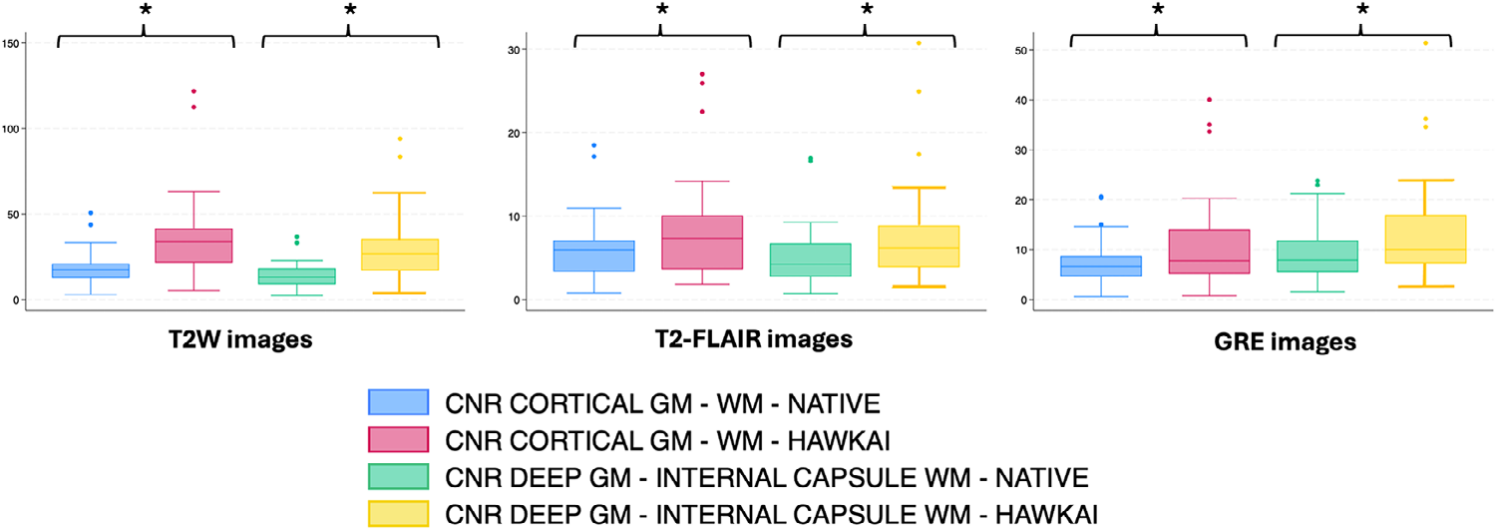
Box and whiskers plots showing the quantitative measurements of CNR on T2W, T2‐FLAIR, and GRE images of 27 brain examinations. GM, Gray matter; WM, White matter. *Statistically significant differences between paired datasets (*p* < .0001 for all tests).

For all contrasts tested (T2W, T2‐FLAIR, and GRE), the SNRs of cortical gray matter, subcortical white matter, deep gray matter, and internal capsule were significantly higher on AI‐treated images compared with native images (*p* < .0001, Figure 2). Similarly, for all contrasts tested, the CNRs between cortical gray and white matter as well as between deep gray matter and internal capsule were significantly higher on AI‐treated images compared with native images (*p* < .0001, Figure 3). The improvement in SNR ranged between 88.1% and 94.4% for T2W images, 34.9% and 40.7% for T2‐FLAIR images, and 38.1% and 41.8% for GRE images. The improvement in CNR ranged between 99.7% and 105.2% for T2W images, 44.4% and 48.6% for T2‐FLAIR images, and 37.7% and 45.7% for GRE images (Table 2).

**TABLE 2:** Average change and 95% confidence Intervals of measured SNR and CNR between AI‐treated images and native images in 27 dogs and cats.

	SNR cortical GM	SNR subcortical WM	SNR deep GM	SNR internal capsule WM	CNR cortical GM—subcortical WM	CNR deep GM—internal capsule WM
T2W	+91.06% [75.27–106.84]	+88.13% [71.82–104.44]	+94.39% [80.01–108.76]	+91.62% [77.22–106.03]	+99.74% [82.90–115.58]	+105.19% [89.07–121.31]
T2‐FLAIR	+35.66% [26.63–44.69]	+34.91% [26.04–43.77]	+40.45% [30.77–50.13]	+40.67% [30.18–51.16]	+48.55% [22.04–75.07]	+44.35% [33.55–55.17]
GRE	+38.07% [27.40‐48.73]	+38.31% [28.10–48.53]	+41.81% [29.87–53.76]	+41.16% [29.47–52.83]	+37.72% [22.65–52.80]	+45.74% [30.70–60.79]

### 3.3 Qualitative Analysis

**FIGURE 4:**
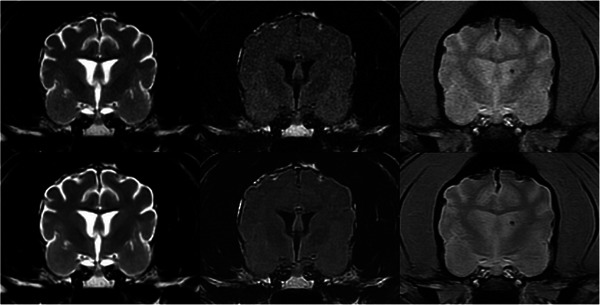
Transverse T2W (left), T2‐FLAIR (middle), and GRE (right) brain MRI images in a 13‐year‐old standard poodle. Native images are at the top, and corresponding AI‐treated images are at the bottom. For this patient, the increase in SNR on AI‐treated images ranged between 77% and 109% for the T2W images, 24.6% and 37.3% for the T2‐FLAIR images, and 61.9% and 84.7% for the GRE images. This patient received higher (i.e., better) coarseness, contrast, and overall quality scores for the AI‐treated images than the native images. A left‐sided microbleed is visible on the GRE images. Slice thickness = 3 mm, interslice gap = 0.5 mm, FOV = 14 cm; T2W image parameters: TR = 3665 ms, TE = 100 ms, NEX = 3; T2‐FLAIR image parameters: TR = 8962 ms, TE = 146 ms, TI = 2376 ms, NEX = 2; GRE image parameters: TR = 717 ms, TE = 15 ms, Flip angle = 20°, NEX = 2.

**FIGURE 5:**
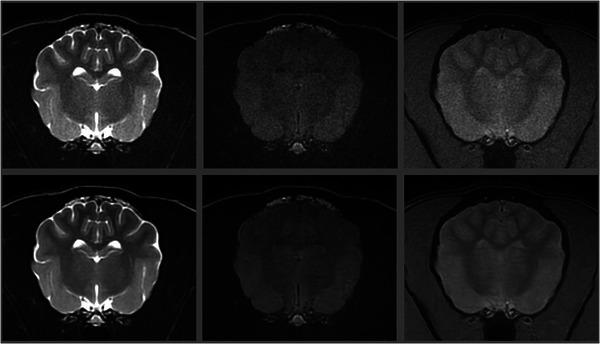
Transverse T2W (left), T2‐FLAIR (middle), and GRE (right) brain MRI images in a 5‐year‐old male castrated Shi Tzu. Native images are at the top, and corresponding AI‐treated images are at the bottom. All three observers assigned higher (i.e., better) quality scores for the AI‐treated images than the native images for T2W and GRE series. For T2‐FLAIR, all three observers assigned the images a similar score of 1 for coarseness, contrast, and overall quality for both the AI‐treated and native images. Slice thickness = 3 mm, interslice gap = 0.5 mm, FOV = 10 cm; T2W image parameters: TR = 3615 ms, TE = 101.7 ms, NEX = 3; T2‐FLAIR image parameters: TR = 8975 ms, TE = 147.4 ms, TI = 2,267 ms, NEX = 2; GRE image parameters: TR = 666.7 ms, TE = 15 ms, Flip angle = 20°, NEX = 2.

Sixty brain scans (30 native sets and 30 AI‐treated sets) were evaluated by the three observers in random order over two months. The median scores and range for each observer and image type are summarized in Table 3. Most median scores were higher on AI‐treated images than the native images (Figure 4). In all but five score categories (three of them being T2‐FLAIR image series, Figure 5), there was a statistically significant difference between the AI‐treated images and the native images (*p* < .05).

**TABLE 3:** Median [range] scores of perceived image quality for native and AI‐treated T2W, T2‐FLAIR, and GRE images for each observer.

Image contrast	Observer	Image characteristics	Native	AI‐treated
T2W images	Observer 1	Coarseness	2 [1–2]^*^	3 [2–3]^*^
		Contrast	2 [1–3]^*^	3 [1–3]^*^
		Overall quality	2 [1–3]^*^	3 [2–3]^*^
	Observer 2	Coarseness	2 [1–3]^*^	3 [2‐3]^*^
		Contrast	2 [2–3]^*^	3 [2‐3]^*^
		Overall quality	2 [1–3]^*^	3 [2‐3]^*^
	Observer 3	Coarseness	2 [1–2]^*^	3 [2‐3]^*^
		Contrast	3 [1–3]	3 [2‐3]
		Overall quality	2 [1–2]^*^	3 [2‐3]^*^
T2‐FLAIR images	Observer 1	Coarseness	1 [0–1]^*^	2 [1‐3]^*^
		Contrast	1 [1–2]^*^	2 [1‐3]^*^
		Overall quality	1 [0–2]^*^	2 [1‐3]^*^
	Observer 2	Coarseness	1 [0–2]^*^	1 [1–3]^*^
		Contrast	2 [1–2]	2 [0–3]
		Overall quality	2 [1–2]	2 [1–3]
	Observer 3	Coarseness	1 [1–2]^*^	2 [1–3]^*^
		Contrast	1.5 [1–3]	2 [1–3]
		Overall quality	1 [1–2]^*^	2 [1–2]^*^
GRE images	Observer 1	Coarseness	1 [1–2]^*^	2 [1–3]^*^
		Contrast	2 [1–2]^*^	2.5 [1–3]^*^
		Overall quality	2 [1–2]^*^	2 [1–3]^*^
	Observer 2	Coarseness	2 [1–3]	2 [1–3]
		Contrast	2 [1–3]^*^	3 [1–3]^*^
		Overall quality	2 [1–3]^*^	3 [1–3]^*^
	Observer 3	Coarseness	2 [1–2]^*^	2 [2–3]^*^
		Contrast	2.5 [1–3]^*^	3 [2–3]^*^
		Overall quality	2 [1–2]^*^	2 [2–3]^*^

* Statistically significant differences between paired datasets (paired Wilcoxon sign‐ranked test).

## 4 Discussion

In this method comparison study, we evaluated a recently developed denoising Deep Learning‐based algorithm explicitly created for veterinary MRI, which used a training dataset of canine and feline clinical MRI images obtained on a variety of low‐ and high‐field MRI scanners. This algorithm works on DICOM datasets (i.e., image space) instead of the raw data (i.e., k‐space) and, therefore, can be applied to any image regardless of the MRI scanner manufacturer.

We conducted a quantitative and qualitative comparison using actual clinical brain MRI scans in dogs and cats. The quantitative analysis demonstrated a consistent improvement of SNR and CNR on AI‐treated images compared with the native images. This indicates that noise reduction with HawkAI increased SNR without reducing image contrast. The improvement in SNR (88.1–94.4%) and CNR (99.7–105.2%) on T2W images was on par with the improvement reported in a quantitative study using a vendor‐specific (AiCE, Canon) denoising deep‐learning reconstruction algorithm (SNR improvement of 54–114% and CNR improvement of 114–117%) [28]. The improvement in SNR and CNR for T2‐FLAIR and GRE images in our study was substantial but more modest, which may be a future avenue of improvement as these types of images are typically more sensitive to signal starvation in practice.

The blinded qualitative analysis corroborated the visual impact of these improvements, although the subjective median scores were not consistently higher for the AI‐treated images. Three of the five score categories without statistically significant differences between native and AI‐treated images were on the T2‐FLAIR image series (Table 3). This reflects the quantitative analysis, with a more modest increase in SNR and CNR for T2‐FLAIR images than for the T2W images (Table 2). This may be due to cognitive bias, leading to systematic low‐grading of T2‐FLAIR images, as these are typically considered by radiologists as grainy, low SNR, and low contrast images compared with other pulse sequences. It is possible that a difference in subjective quality between native and AI‐treated T2‐FLAIR images would have been more clearly observed if pairs of images had been compared side by side, as has been observed in human studies [29]. Still, most median image quality scores on T2‐FLAIR HawkAI‐treated images were 2, compared with a majority of median scores of 1 for the T2‐FLAIR native images (Table 3). These results indicate that noise reduction provided by this algorithm can be detected perceptually by radiologists.

In this study, we focused on verifying the effectiveness of a DICOM‐based noise‐reduction algorithm developed for veterinary patients to improve the quality of standard MRI images obtained with a commercially available MRI scanner. Previous studies in dogs have shown that fewer acquisitions (hence shorter acquisition time) with a proprietary k‐space‐based deep learning noise reduction algorithm can produce images of equivalent or better quality than with a high number of acquisitions [30]. This is a possible use of denoising algorithms in veterinary patients, as anesthesia time for MRI examination is a common clinical concern. The previously mentioned MRI scanner‐specific algorithms (such as AirReconDL by GE Healthcare or DeepResolve by Siemens) were trained and developed on human datasets, and some of them were developed using the K‐space domain, where training inputs were synthetically generated from regular standard‐of‐care MRI images [31].

HawkAI was developed and trained using an extensive dataset of canine and feline paired images—encompassing both high‐field and low‐field MRIs—and purely developed in the image domain on real paired images, which makes it vendor‐agnostic and compatible with images from multiple MRI manufacturers.

There are several potential uses of platform‐agnostic denoising algorithms such as HawkAI in veterinary medicine. For example, they could improve image quality obtained with low‐field MRI scanners, which have inherent low SNRs and are prevalent in veterinary practice worldwide. As has been reported with vendor‐specific proprietary deep‐learning reconstruction tools [28], HawkAI may improve the image quality of 1.5T scanners, comparable to that obtained at 3T, which is less widely available in veterinary medicine and is significantly more expensive. There is potential to increase the diagnostic yield of 1.5T images, such as more confident identification of small lesions that may be obscured by noise, such as tiny lacunar infarcts. Another useful application would be to reduce scan time (e.g., decrease NEX) and apply the algorithm to the noisier and less resolute image and still get a result equivalent to longer pulse sequences, as has been recently shown with vendor‐specific, k‐space‐based deep learning‐based reconstruction algorithms [30].

This study has several limitations. The number of MRI studies included was relatively small; however, the statistical analyses revealed significant differences across the board, with consistent differences observed between native and AI‐treated images. The qualitative (subjective) image quality assessment results confirmed the quantitative (objective) analysis findings, further supporting the effectiveness of the noise reduction method reported herein. The ROIs for quantitative analysis were placed manually, which can lead to measurement errors. However, the propagation tool of the DICOM viewer used ensured a consistent anatomical placement of the ROIs across all types of imaging series for each patient, hence mitigating variability. The objective comparisons were not made side by side for the qualitative analysis to avoid scoring bias; instead, the three radiologists assessed each of the 30 pairs of studies one by one, being blinded to the type of images. There is a possibility of recall bias for cases in which apparent lesions were present; however, this was mitigated by the randomization of the order in which the studies were evaluated and the extended period over which the assessment occurred. We only compared the noise‐reduction algorithm with the native images on our scanner but did not compare it with the vendor‐specific deep‐learning noise reduction solution (Air Recon DL, GE Healthcare) as this option was unavailable on the study scanner. Future studies are necessary to conduct such comparisons. Although the noise‐reduction algorithm can be applied to all pulse sequences (a difference from some scanner‐specific AI solutions, which can only be applied to some pulse sequences), we only evaluated the T2W, T2‐FLAIR, and GRE sequences as they are some of the sequences most affected by noise. Future studies could be conducted to extend this analysis to other pulse sequences. Our study focused on subjective and objective noise, contrast, and image quality assessment on the three pulse sequences tested. However, the impact of the denoising algorithm on the clinical diagnosis was not evaluated and should be the topic of future studies. Finally, the noise‐reduction algorithm was only assessed on images generated by a machine from a single manufacturer, and future studies are necessary to evaluate the method on units from other manufacturers and confirm its applicability across many imaging platforms.

In conclusion, this study supports the use of a DICOM‐based deep learning denoising algorithm to reduce noise in 1.5T MRI canine and feline brain images. It resulted in higher SNRs and CNRs than the original images. In addition, radiologists’ perceived image quality improved, including the subjective noise level (coarseness), perceived contrast between intracerebral structures, and overall image quality.

## List of Author Contributions

**Category 1**

- Conception and design: Mai
- Acquisition of data: Eddin, Mai, Hecht, Paek, Holmes, Dorez, Blanchard
- Analysis and interpretation of data: Mai

**Category 2**

- Drafting the Article: Mai
- Reviewing the article for intellectual content: Mai, Hecht, Paek, Holmes, Dorez, Blanchard, Eddin

**Category 3**

- Final approval of the completed article: Mai, Hecht, Paek, Holmes, Dorez, Blanchard, Eddin

**Category 4**

- Agreement to be accountable for all aspects of the work in ensuring that questions related to the accuracy or integrity of any part of the work are appropriately investigated and resolved: Mai, Hecht, Paek, Holmes, Dorez, Blanchard, Eddin

## Ethics Statement

All owners had signed consent forms to use patient data at admission to the hospital. Therefore, an Institutional Animal Care and Use Committee approval was not necessary.

## Conflicts of Interest

The radiologists performing data analysis in this study (W. M., S. H., M. P., and S. P. H.) have no official affiliation with the company that created the tested deep learning algorithm and did not receive any compensation for their contribution to this study. The authors declare no conflicts of interest.

## Previous Presentation or Publication Disclosure

The findings presented herein were not presented at scientific meetings or published in abstracts.

## Equator Network Disclosure

No EQUATOR network checklist was used.
